# Artificial intelligence facilitates information fusion for perception in complex environments

**DOI:** 10.1016/j.xinn.2025.100814

**Published:** 2025-01-29

**Authors:** Xunpeng Yi, Yong Ma, Yansheng Li, Han Xu, Jiayi Ma

**Affiliations:** 1 Electronic Information School, Wuhan University, Wuhan 430072, China; 2 School of Remote Sensing and Information Engineering, Wuhan University, Wuhan 430079, China; 3 School of Automation, Southeast University, Nanjing 210096, China

## Main text

Multimodal perception is a foundational technology for human perception in complex environments. These environments often involve various interference conditions and sensor technical limitations that constrain the information capture capabilities of single-modality sensors. Multimodal perception addresses these by integrating complementary multisource heterogeneous information, providing a solution for perceiving complex environments. This technology spans across fields such as autonomous driving, industrial detection, biomedical engineering, and remote sensing. However, challenges arise due to multisensor misalignment, inadequate appearance forms, and perception-oriented issues, which complicate the corresponding relationship, information representation, and task-driven fusion. In this context, the advancement of artificial intelligence (AI) has driven the development of information fusion, offering a new perspective on tackling these challenges.^1^ AI leverages deep neural networks (DNNs) with gradient descent optimization to learn statistical regularities from multimodal data. By examining the entire process of multimodal information fusion, we can gain deeper insights into AI’s working mechanisms and enhance our understanding of AI perception in complex environments.

## AI for misalignment information matching

While multimodal sensors provide multisource heterogeneous information for a complete scene representation, their limitations stem from factors like sensor position, angle, and inherent differences in sensor parameters. These factors lead to mismatches in different information, which adversely affect subsequent fusion. Fortunately, AI can learn statistical correspondences from existing multisource heterogeneous data, enabling adaptive matching in new scenarios.^2^ This data-driven learning approach indicates significant potential for robust information matching.

**Figure 1:**
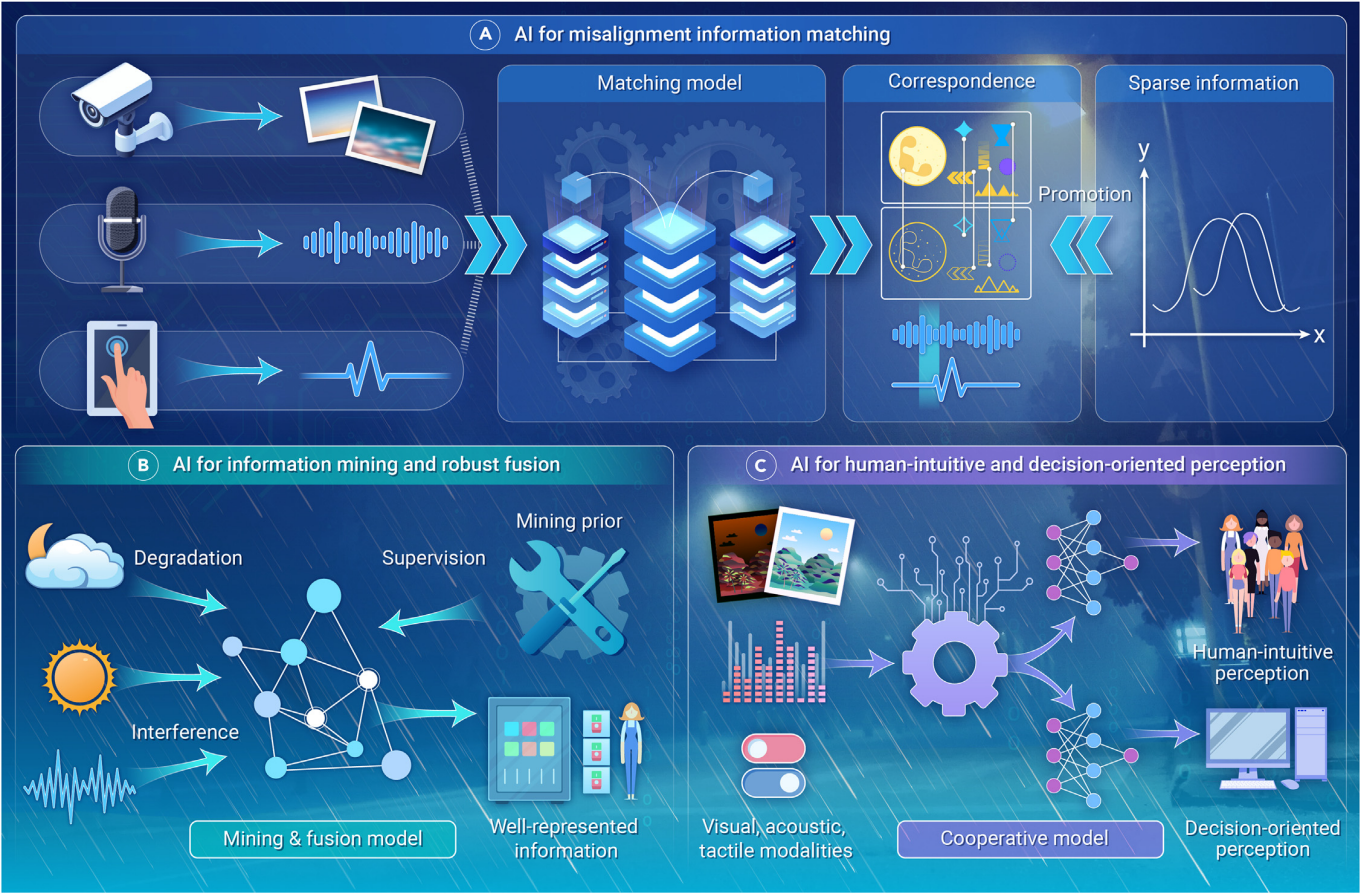
Framework of AI for information fusion in perceiving complex environments

AI identifies correspondences in multisource heterogeneous data through two primary steps: feature extraction and correspondence regression (Figure 1A). These steps are closely interconnected, with effective feature extraction serving as a crucial prerequisite for accurate correspondence regression. However, the apparent heterogeneity in multisource data poses difficulties in extracting features that are conducive to regression. By discarding manually designed feature extractors, AI leverages DNNs to adaptively establish modality-invariant and intrinsic consistency presentations across different modalities. In terms of implementation, this can involve either explicit constraints or implicit implementations in an end-to-end manner. Naturally, regardless of the specific implementation, their ultimate objective is to extract modality-invariant and intrinsic consistency features. These features serve as the basis for subsequent regression of correspondences. Moreover, AI utilizing DNNs as regressors is a critical improvement. It frames correspondence finding as a function mapping problem, emphasizing a coarse-to-fine, multilevel approach. By integrating dense matching, semi-dense matching, or other parameter estimation methods, DNNs can regress discrepancies in multisource heterogeneous data, forming the basis for correction and subsequent fusion.

The aligned data often exhibit apparent local sparsity, particularly evident in the sparse edge gradients of multisource heterogeneous images, where fully corresponding heterogeneous images do not contain overlapping object edges. By leveraging this characteristic through AI, the reliability of misalignment information matching is further enhanced.

## AI for information mining and robust fusion

Complex environments may cause the loss and occlusion of information within a single modality, resulting in incomplete representations. This can exacerbate the imbalance in fused appearance, thereby compromising the effect of fusion. To some extent, this hampers human perception. Therefore, it is crucial to address the issue of information appearance, mine non-prominent information, and achieve robust fusion to ensure effective perception.

AI can derive information mining priors and supervision from well-represented data (Figure 1B). It may consist of precisely paired data generated by advanced instruments at the same timestamp, suitable for supervised learning. Alternatively, it may also include unpaired natural data representing well-characterized statistical distributions, which can be used for unsupervised or weakly supervised learning, such as adversarial or contrastive learning. AI has the capability to learn effective priors from diverse datasets, enabling robust generalization across various scenarios. Naturally, the construction of high-quality data plays a critical role in the training process. It directly influences the learning direction of AI, determining whether the resulting models are comprehensive or biased.

Beyond data construction, the interaction for leveraging multisource information is also critical, involving the structural design of fusion. Given the scene-relatedness of multisource data, the fusion module can integrate features from other modalities to improve information gain.

Moreover, information fusion can benefit from leveraging diverse heterogeneous data. Beyond the visual modality, this includes other modalities, such as text, auditory, and tactile sensing in a broader sense. AI aims to integrate these diverse data sources by leveraging their complementarity to generate a unified representation for further processing. A practical example involves equipping intelligent robots with multisensor systems, such as visual, temperature, pressure, and auditory sensors.^3^ Through robust fusion using DNNs, a comprehensive representation is formed. This representation then facilitates the robot’s decision-making process or enhances human perception. Similarly, in biomedical applications, mass spectrometry imaging (MSI) is an analytical technique used to investigate the distribution of biomolecules and their spatial arrangement. However, MSI only achieves resolution at the single-cell level.^2^ When integrated with MRI data, it can achieve cell-type-specific resolution at the tissue level across the entire brain. In this process, the deep coupling of multisource heterogeneous features further enhances information fusion.

## AI for human-intuitive and decision-oriented perception

Information fusion for perception can be applied to both human-intuitive subjective tasks and machine decision-oriented tasks.^4^ At the macro level, the common objective is to create a holistic scene representation and then execute corresponding guided tasks. At the micro level, human-intuitive subjective tasks focus on attributes like intensity, texture, and color in the visual modality; meaning in textual or auditory modalities; and actions in the tactile modality. In contrast, machine decision-oriented tasks focus on attributes such as object coherence and semantic environmental information.

AI can leverage the unity of human-intuitive and decision-oriented objectives to build a collaborative optimization model (Figure 1C). A central challenge lies in integrating fusion features from human-intuitive and decision-oriented perception tasks, maximizing their shared characteristics and mutual support while minimizing conflicts arising from their differences. One potential approach is to adopt a multiencoder, fusion layer, and multidecoder architecture. The encoder is dedicated to extracting information for subsequent fusion, the fusion layer emphasizes feature interaction to achieve a holistic scene representation, and the decoder serves specific requirements of multiple tasks. Furthermore, by integrating gradient optimization strategies, the collaborative optimization of multiple tasks is balanced to achieve a synthetically optimal solution.

Given its strong modeling capabilities, AI is capable of coordinating multioriented perception in complex environments. Moreover, it enables the mutual collaboration of multiple orientations and activates the potential for task reinforcement, thereby further enhancing the comprehensiveness of perception.

## Future trends and challenges

With technological advancements, the entire chain—from data acquisition to algorithm models and decision services—has made significant progress. Exploring the untapped potential of AI for information fusion in perceiving complex environments is highly significant. On the one hand, perceptual performance of current AI in complex environments remains limited by the scale and richness of available data. However, the emergence of large models, such as ChatGPT, offers promising avenues for overcoming this bottleneck.^5^ These large models are trained on richer data, equipping them with more comprehensive information extraction and fusion capabilities. And the incorporation of their prior knowledge into models further enhances their fusion performance. Additionally, advanced network architectures, such as Transformer, provide the necessary learning capabilities for models to be effectively trained on large datasets. Data and models complement each other and collaboratively improve AI performance. On the other hand, human-interactive AI represents a promising avenue for future developments in perception applications within complex environments. Whether the task is human-intuitive subjective or machine decision-oriented, a human-centered ideology is crucial. Providing interactive interfaces, such as text or voice inputs, enables more flexibility of AI systems. These elements contribute to AI to achieve more accurate and requirement-compliant fusion perception.

Naturally, developments are accompanied by challenges. Existing AI systems heavily rely on data-driven training. The alignment of cross-modal data necessitate learning from pre-matched data. However, the acquisition of multisource registered data and human-instructed data is highly costly and resource intensive. Utilizing the vast amount of publicly accessible internet data could address a substantial part of these. However, this raises a new challenge: how to safeguard privacy while automatically leveraging these data to enhance model performance and extend applications. Leveraging federated learning and privacy-preserving data cleaning strategies could offer potential solutions to this challenge. Furthermore, with regard to model outputs, the development of more diverse representation forms and the support of a wider array of decision-making tasks represent key challenges for future research. Although opportunities and challenges are equally prominent, they do not diminish our confidence in the future of AI in complex environment perception.

## Acknowledgments

This work was supported by the 10.13039/501100001809NSFC (U23B2050 and 62276192).

## Declaration of interests

The authors declare no competing interests.
